# Enhancing Cartilage Metabolism in Rats through a Novel Thermal Stimulation Technique with Photosensitizers

**DOI:** 10.3390/ijms25126728

**Published:** 2024-06-19

**Authors:** Ryota Cha, Shuji Nakagawa, Yuji Arai, Atsuo Inoue, Naoki Okubo, Yuta Fujii, Kenta Kaihara, Kei Nakamura, Tsunao Kishida, Osam Mazda, Kenji Takahashi

**Affiliations:** 1 Department of Orthopaedics, Graduate School of Medical Science, Kyoto Prefectural University of Medicine, Kawaramachi-Hirokoji, Kamigyo-ku, Kyoto 602-8566, Japan; r-cha@koto.kpu-m.ac.jp (R.C.);; 2 Department of Sports and Para-Sports Medicine, Graduate School of Medical Science, Kyoto Prefectural University of Medicine, Kawaramachi-Hirokoji, Kamigyo-ku, Kyoto 602-8566, Japan; 3 Department of Immunology, Graduate School of Medical Science, Kyoto Prefectural University of Medicine, Kawaramachi-Hirokoji, Kamigyo-ku, Kyoto 602-8566, Japan

**Keywords:** HSP70, osteoarthritis, thermotherapy

## Abstract

Although the moderate thermal stimulation of articular cartilage exerts chondroprotective effects, it is difficult to effectively heat deep articular cartilage with conventional methods. Photosensitizers increase the ambient temperature using near-infrared (NIR) radiation, which has high tissue permeability. We hypothesized that the intra-articular administration of photosensitizers and NIR irradiation would exert a greater heating effect on articular cartilage. We aimed to evaluate the heating effect of this method on cultured chondrocytes and rat knee cartilage. In vitro, we irradiated a photosensitizer-containing medium with NIR and measured changes in the medium temperature, cytotoxicity, and gene expression of heat shock protein (*HSP*) *70* and aggrecan (*ACAN*). In vivo, the knee joints of rats treated with photosensitizers were irradiated with NIR, and changes in intra-articular temperature and gene expression were measured, alongside histological analysis. The results showed that the medium and intra-articular temperature were raised to approximately 40 °C with no apparent disruption to articular cartilage or the immunohistochemically enhanced staining of HSP70 in chondrocytes. The gene expression of *HSP70* and *ACAN* was increased in both cultured and articular cartilage. In summary, this method can safely heat joints and enhance cartilage metabolism by inducing HSP70 expression in articular cartilage. It presents a new hyperthermia therapy with effective cartilage protection.

## 1. Introduction

Osteoarthritis (OA) is a chronic degenerative disease causing articular cartilage breakdown, leading to significant mobility impairment, pain, and reduced activities of daily living (ADL) in the elderly [1,2]. Its prevalence is expected to continue to rise due to increasing life expectancies [3]. Treatment for OA begins with a combination of conservative therapies, including lifestyle guidance, medication, and physical therapy [4]. Thermotherapy is widely employed in clinical practice for its effectiveness in relieving pain, improving tissue flexibility, and improving blood flow [5]. Many reports have shown that heating articular cartilage suppresses cartilage degeneration. Heat shock protein (HSP) 70, which is induced in chondrocytes by thermal stimulation, is known to suppress the apoptosis of chondrocytes and increase the expression of proteoglycans such as aggrecan (ACAN) and type II collagen (Col II) at the moderate temperature of about 40 °C [6]. In animal experiments, thermal stimulation using microwaves (MWs) has been shown to enhance cartilage metabolism in rabbit knee joints by increasing HSP70 expression and to inhibit the progression of OA in rats [5,7]. However, the effects of hot packs and paraffin baths, which are widely used in clinical practice as thermotherapy, are limited to the subcutaneous region [8], and MW also absorbs most of its energy in the superficial layer, making it difficult to effectively heat the deep articular cartilage [9]. In order to maximize the cartilage protective effect of hyperthermia, it is desirable to develop new thermal stimulation techniques that can efficiently heat deep joints.

Photosensitizers convert light energy into molecular vibration energy when exposed to near-infrared (NIR) radiation of a specific wavelength, transferring heat energy to the surrounding environment. Photothermal therapy (PTT) using photosensitizers has been attracting attention as a cancer treatment method in recent years [10,11,12]. It entails a photosensitizing substance administered into the lesion and irradiated with highly bio-permeable NIR, which causes a localized increase in temperature, producing an anti-tumor effect. The temperature within the target and its duration can be controlled by adjusting the irradiation of light rays. We hypothesized that administering a photosensitizer with heat-generating properties into the joint and externally irradiating it with NIR could uniformly heat the joint from within, offering an effective hyperthermia therapy with effective cartilage protection. Based on the above background, we aimed to evaluate the heating effect of photosensitizers on cultured chondrocytes and articular cartilage and to develop a new thermal stimulation technique.

## 2. Results

### 2.1. In Vitro Studies

#### 2.1.1. Heating Effects of Indocyanine Green (ICG) and NIR on the Medium

**Figure 1:**
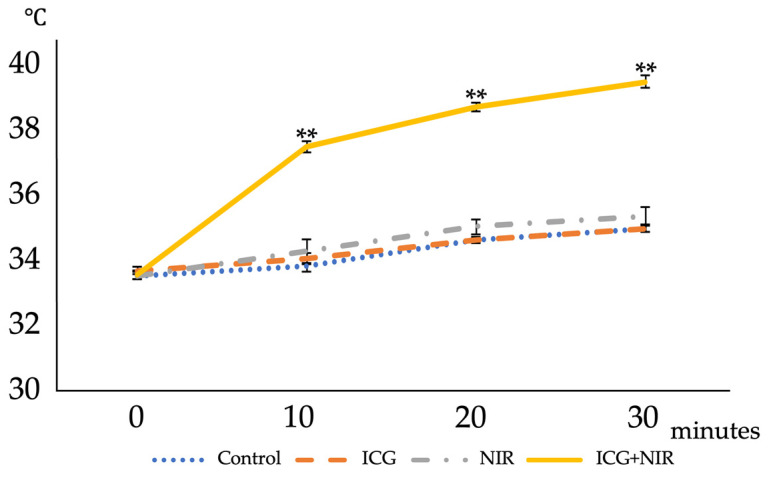
Change in medium temperature with NIR with or without NIR irradiation and ICG. Cultured chondrocytes were divided into four groups according to the presence or absence of NIR irradiation and the addition of ICG to the culture medium, and the temperature change during the first 30 min of irradiation was recorded. Each value represents the mean ± SD (n = 6). ** *p* < 0.01.

The heating effect of NIR irradiation on ICG-added medium was evaluated by measuring the change in medium temperature over time (Figure 1). The change in medium temperature over 30 min was 33.5 ± 0.12 °C to 34.9 ± 0.1 °C for the control group, 33.6 ± 0.12 °C to 35.0 ± 0.11 °C for the ICG group, and 33.5 ± 0.12 °C to 35.3 ± 0.27 °C for the NIR group. On the contrary, in the ICG+NIR group, the medium temperature increased from 33.5 ± 0.12 °C to 39.5 ± 0.2 °C, which was significantly higher than that of the other groups (*p* < 0.01).

#### 2.1.2. Effects of ICG and NIR on LDH Release in Chondrocytes

**Figure 2:**
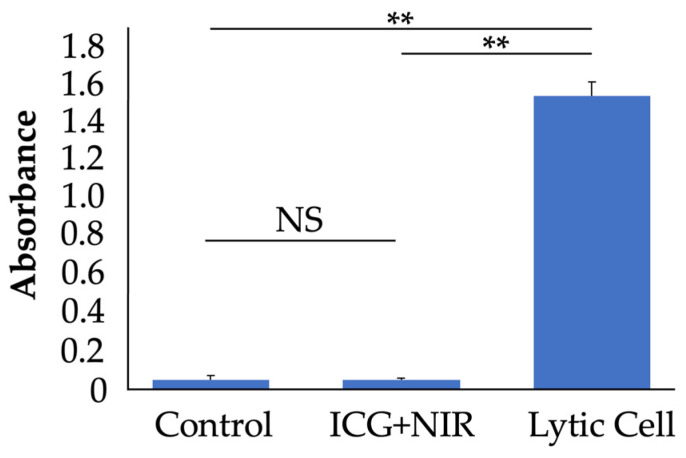
Cytotoxicity of ICG+NIR treatment was evaluated. Absorbance (490 nm) of each group, measured by LDH release assay. Control group, no treatment; ICG+NIR group; ICG was added to the culture medium and irradiated with NIR for 30 min; lytic cell group, chondrocytes lysed with Lysis Solution (n = 9). ** *p* < 0.01.

Cytotoxicity to chondrocytes was evaluated using an LDH release assay (Figure 2). Cultured chondrocytes were divided into three groups: a no-treatment group (n = 9), a group with ICG added to the medium and irradiated with NIR (ICG+NIR group, n = 9), and a group with cell lysis (lytic cell group, n = 9). LDH release was significantly lower in the control and ICG+NIR groups compared to the lytic cell group, at approximately 3% (*p* < 0.01). There was no significant difference in LDH release between the control and ICG+NIR groups.

#### 2.1.3. Effects of ICG and NIR on Gene Expression in Chondrocytes

**Figure 3:**
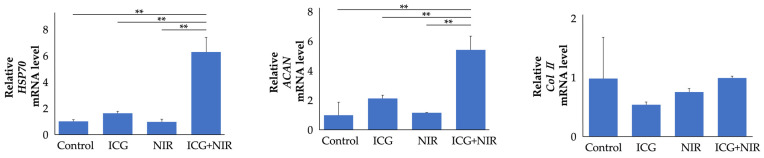
Gene expression after ICG and/or NIR treatment on chondrocytes. After 6 h, total RNA was extracted from cells. mRNA levels in chondrocytes were analyzed through real-time RT-PCR analysis (n = 9). ** *p* < 0.01.

Gene expression was evaluated 6 h after ICG and/or NIR treatment (Figure 3). Cultured cells were divided into four groups: control, ICG, NIR, and ICG+NIR (each n = 9). The *HSP70* and *ACAN* gene expression levels in the chondrocytes of the ICG+NIR group had increased significantly compared to the other groups (mean values of *HSP70* 6.3-fold and *ACAN* 5.4-fold compared to the control group, *p* < 0.01). *Col II* gene expression levels were not significantly different compared to the other groups.

#### 2.1.4. Role of HSP70 in ICG and NIR Response in Chondrocytes

**Figure 4:**
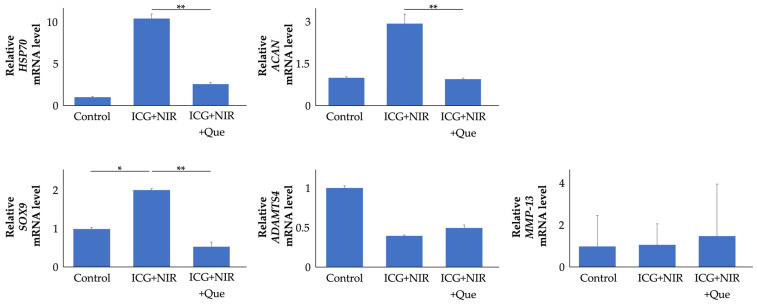
Gene expression in the *HSP70* suppression test using Que in ICG+NIR treatment. After 6 h, total RNA was extracted from cells. mRNA levels in chondrocytes were analyzed through real-time RT-PCR analysis (n = 9). * *p* < 0.05, ** *p* < 0.01.

*HSP70* suppression experiments were performed using quercetin (Que) (Figure 4). *HSP70* gene expression in Que-treated cells was reduced to 25% compared to that in ICG+NIR cells (*p* < 0.01). *ACAN* gene expression in Que-treated cells was reduced to 32% compared to that in ICG+NIR cells (*p* < 0.01). SRY-box (*SOX*) *9* gene expression levels in the chondrocytes of the ICG+NIR group were increased significantly—2.0-fold, on average—compared to the control group. A disintegrin and metalloproteinase with thrombospondin motifs (*ADAMTS*) *4* and matrix metalloproteinase (*MMP*)*-13* gene expression levels were not significantly different in any group.

### 2.2. In Vivo Studies

#### 2.2.1. Heating Effects of ICG and NIR on Rat Knee Joints

**Figure 5:**
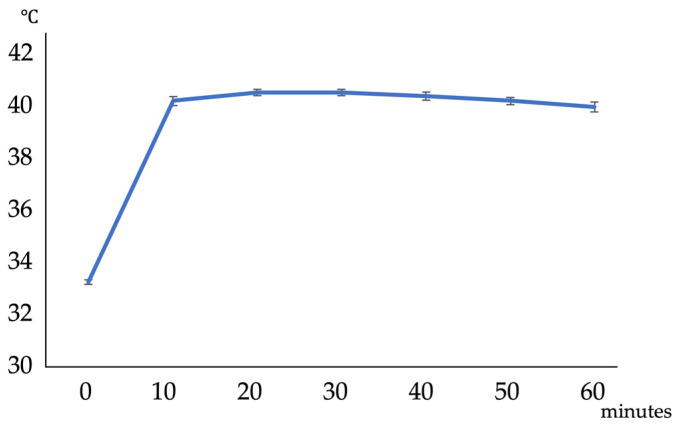
Successive changes in intra-articular temperature following ICG intra-articular injection and NIR irradiation. Temperature changes during the first 60 min of irradiation were recorded. Each value represents the mean ± SD (n = 5).

NIR irradiation was performed on the knee joints of rats injected with ICG intra-articularly, and changes in intra-articular temperature were measured (Figure 5). The intra-articular temperature increased from 33.2 ± 0.09 °C to 40.1 ± 0.15 °C 10 min after the start of irradiation and then slowly decreased to 39.9 ± 0.19 °C at the end of 60 min of irradiation. No complications, such as skin redness, swelling, or knee joint contracture, were observed in the rats.

#### 2.2.2. Effects of ICG and NIR on Articular Cartilage Tissue

**Figure 6:**
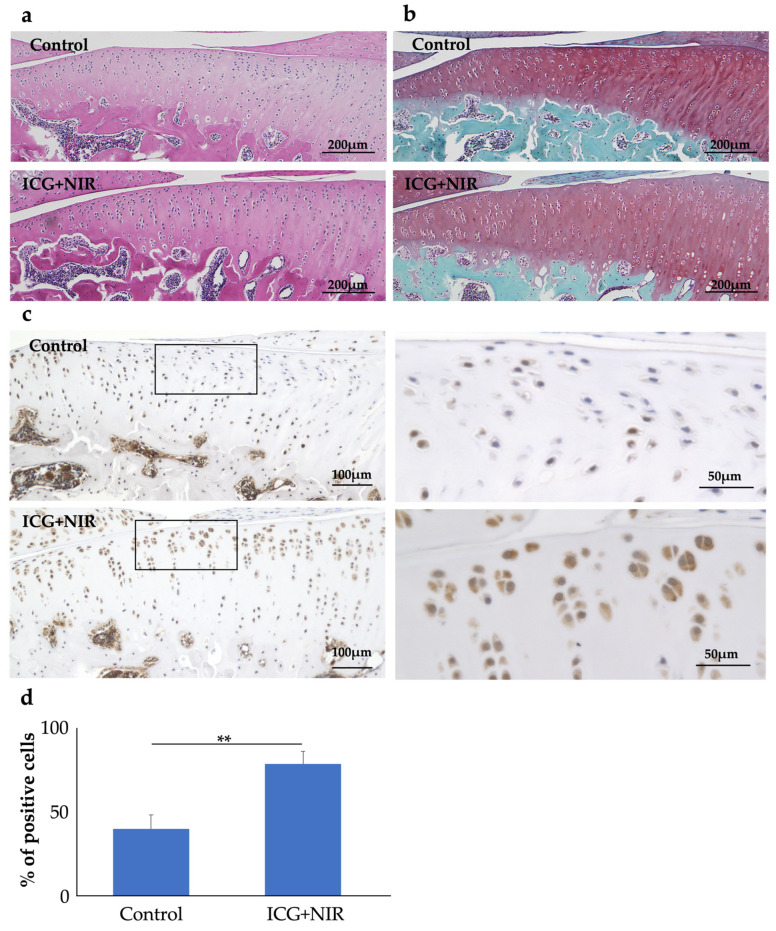
Representative photographs of HE staining (**a**), Safranin O staining (**b**), and HSP70 immunostaining (**c**) sagittal sections and % of positive staining cells (**d**) from rat knee joints collected after 8 h of NIR irradiation. ** *p* < 0.01.

Eight hours after the end of NIR irradiation, the knee joint was collected and evaluated histologically (Figure 6). Rats were divided into two groups: ICG injected into the knee joint with NIR irradiation (ICG+NIR group, n = 5) and phosphate-buffered saline (PBS) injected into the knee joint without NIR irradiation (control group, n = 5). HE staining (Figure 6a) and Safranin O staining (Figure 6b) showed no tissue damage and no difference in staining in either group. HSP70 immunostaining (Figure 6c) showed that mainly, superficial chondrocytes were more strongly stained in the ICG+NIR group than in the control group (the positive cell counts were 24.0% vs. 82.2%, Figure 6d).

#### 2.2.3. Effects of ICG and NIR on Gene Expression in Articular Cartilage

**Figure 7:**
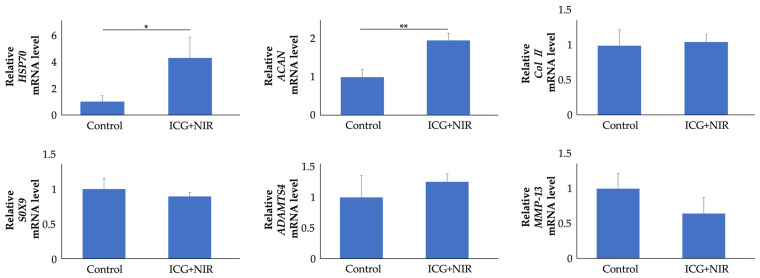
Gene expression in rat knee cartilage after ICG+NIR treatment. Total RNA was extracted from articular cartilage collected from rats 8 h after the end of treatment. mRNA levels in chondrocytes were analyzed through real-time RT-PCR analysis (n = 5). * *p* < 0.05, ** *p* < 0.01.

Articular cartilage was harvested 8 h after the end of NIR irradiation, and gene expression was evaluated (Figure 7). *HSP70* gene expression levels in the ICG+NIR group were significantly elevated—4.2-fold, on average—compared to the control group (*p* < 0.01). The *ACAN* gene expression levels in the ICG+NIR group were also significantly elevated—2.0-fold, on average—compared to the control group (*p* < 0.05), while other gene expression levels were not significantly different.

## 3. Discussion

We evaluated the effects of a thermal stimulation technique using photosensitizers and NIR irradiation on chondrocytes and articular cartilage. In vitro, this method can apply moderate thermal stimulation, at about 40 °C, to the culture medium of chondrocytes, and it was found that the enhancement of *HSP70* expression heightened the cartilage metabolic activity. In vivo, a moderate temperature increase in the knee joints of rats was found to increase HSP70 expression and cartilage metabolic activity.

Thermotherapy is widely used in clinical practice as a physical therapy for OA and has been shown to reduce pain by increasing the pain threshold of soft tissues, increase the elasticity of collagen fibers, relax muscles due to decreased sensitivity to muscle spindle stretch, and increase local blood flow [13,14,15]. In vitro, heat stimulation of chondrocytes at an appropriate level (39–41.8 °C for 15–30 min) positively affected cell viability and aggrecan metabolism in chondrocytes [6], and in vivo, increasing the intra-articular temperature to approximately 40 °C suppressed OA [7]. It has been reported that heating the articular cartilage inhibits cartilage degeneration. Conventional thermal stimulation techniques such as bathing, hot packs, and MW can easily heat the entire joints of small-to-medium-sized animals such as rabbits, but it is difficult for thermal energy to reach the cartilage located deep in human-sized joints [8,16,17]. In order to maximize the cartilage-protective effect of hyperthermia in clinical practice, it is essential to develop new thermal stimulation techniques that can efficiently heat deep joints. On the contrary, PTT is a method of providing thermal stimulation by generating thermal energy from deep tissue, and this is being attempted in hyperthermia therapy for cancer through the application of the properties of photosensitizers to absorb light energy and convert it into thermal energy for the surrounding area [18]. Photosensitizers enter an excited state by absorbing light energy. Subsequently, non-radiative relaxation is triggered, and the increased kinetic energy releases thermal energy into the surroundings [19]. In addition, PTT uses light rays with wavelengths with high tissue permeability located in the NIR region, which reduces the impairing effect on tissues other than the target tissue where the photosensitizing substance is administered and allows the light energy to reach the deeper-located target tissue with less attenuation [20]. In this study applying this PTT technique, ICG was administered into the joint as a photosensitizing substance and irradiated with near-infrared radiation, a highly tissue-permeable NIR, from the outside, to safely and effectively heat the inside of the joint.

The degree of PTT heating is affected by the type and concentration of photosensitizing substances, the amount of light irradiation, and the duration of irradiation, so it is important to set the conditions for proper heating inside the joint [21]. The performance of photosensitizers also affects the amount of thermal energy generated [22,23]. Gold- and carbon-based nanoparticles, among other photosensitizers, exhibit high photothermal conversion efficiency. However, concerns about their biocompatibility arise from their low degradation rate in living organisms, rendering them currently unsuitable for biological applications [22,24]. On the contrary, ICG, although inferior in photothermal conversion efficiency, is widely used for liver function tests, retina imaging, and fluorescence imaging of tumors in addition to PTT, and there are few concerns regarding its biocompatibility and safety [25,26,27,28]. Therefore, in this study, ICG was used as a photosensitizer to provide thermal stimulation by external NIR irradiation. ICG concentration, irradiation dose, and irradiation time were examined in preliminary experiments. In vitro, 0.05 g/L of ICG was added to the culture medium and irradiated with NIR at 60 mW for 30 min. In vivo, 50 μL of 2.5 g/L ICG solution was injected into the rat knee joint and irradiated with NIR at 185 mW for 1 h. As a result, it was possible to heat ICG-infused medium and rat knee joints to the target temperature by NIR irradiation without obvious disruption to the surrounding tissues and articular cartilage.

HSP70 is thought to play a critical role in the cartilage-protective effects of hyperthermia. HSP is the generic name for a family of proteins that are synthesized in cells after various stress loads, including mechanical and thermal stress. HSP70 expression in chondrocytes increases with OA severity, and its activation inhibits cell death while boosting the production of proteoglycans, such as ACAN, leading to protective effects in cartilage [29,30,31,32,33,34,35]. In addition, the induction of HSP70 in the knee cartilage of animal OA models reportedly inhibits OA progression [36,37], and HSP70 plays an important role in articular cartilage homeostasis. Moderate thermal stress applied to the articular cartilage effectively induces HSP70 in chondrocytes, promoting cartilage protection [5,6,7,30,38]. In this study, HSP70 was efficiently expressed in chondrocytes, and articular cartilage and cartilage metabolism were enhanced by moderate thermal stimulation with photosensitizers and NIR irradiation. We also used Que, a flavonoid that inhibits HSP70, to confirm whether HSP70 mediates this effect in vitro. Que significantly suppressed *HSP70* expression and reversed the effect of heat stimulation on cartilage metabolism. In summary, these results suggest that moderate thermal stimulation with ICG and NIR irradiation effectively expresses HSP70 in chondrocytes and articular cartilage, thereby increasing cartilage metabolism.

As a mechanism by which HSP70 exerts its chondroprotective effect, HSP70 reportedly controls the expression of SOX9, an important anabolic factor in cartilage, and ADAMTS and MMPs, which are representative catabolic factors [32,34,35,37,39,40,41]. In the present study, *SOX9* expression was also increased in chondrocytes in which *HSP70* expression was increased by thermal stimulation in vitro, and the increased *SOX9* expression was cancelled when *HSP70* expression was suppressed by Que, suggesting that HSP70 regulates SOX9 expression and exerts a cartilage protective effect in this thermal stimulation. In contrast, *ADAMTS4* and *MMP-13* expression was not altered. As normal chondrocytes and rats were used in the present study, it is possible that their expression was low [42,43] and that a decrease in expression could not be detected following stimulation.

The present study had a few limitations. First, despite *SOX9* being upregulated with *HSP70* expression via thermal stimulation in vitro, *SOX9* expression was not upregulated in vivo. Second, the present study examined the outcome of a single thermal stimulation, with the long-term effects on cartilage tissue and osteoarthritis undetermined. Third, while successful in heating rat knee joints, the efficacy of this method in reaching the deeper regions of larger human joints remains uncertain.

## 4. Materials and Methods

### 4.1. In Vitro Studies

#### 4.1.1. Chondrocyte Isolation and Culture

Four male Wistar rats aged six weeks (Shimizu Laboratory Supplies, Kyoto, Japan) were anesthetized deeply with pentobarbital and euthanized, and articular cartilage was aseptically collected from the knee, hip, and shoulder joints. The isolated chondrocytes were cultured in 75 cm^2^ flasks (Corning Incorporated, Corning, NY, USA) at 37 °C in 5% CO_2_/95% humidified air (standard conditions) in Dulbecco’s modified Eagle’s medium (DMEM; Nacalai Tesque, Kyoto, Japan) supplemented with 10% fetal bovine serum (Biosera, France) and 1% penicillin–streptomycin mixed solution (complete DMEM; Nacalai Tesque, Kyoto, Japan).

#### 4.1.2. ICG Addition and NIR Irradiation

ICG (Daiichi Sankyo Co., Ltd., Tokyo, Japan) was used as the photosensitizer. ICG has an absorption range from 600 to 900 nm, with an absorption peak of 790 to 805 nm [44,45], and NIR at 800 nm, which is often used in clinical practice, was used.

Cultured rat chondrocytes were treated with trypsin/ethylenediamine triacetic acid (Nacalai Tesque, Kyoto, Japan), resuspended in complete medium, and seeded in a six-well culture plate (Greiner Bio-One, Kremsmünster, Australia) at a density of 1 × 10^5^ cells. At 80% confluence, cells were divided into four groups: control, ICG, NIR, and ICG+NIR. Irradiation intensity and ICG concentration were preliminarily studied. The medium was supplemented with ICG at concentrations of 0.01–2.5 g/L and irradiated for 30 min, with the irradiation intensity adjusted to 0–80 mW. The final medium temperature increased proportionally with increasing concentration and intensity. In subsequent experiments, the ICG concentration was set at 0.05 g/L and the irradiation intensity was set at 60 mW. The medium was changed to DMEM or DMEM containing 0.05 g/L ICG, and chondrocytes were cultured for 30 min under standard conditions. Using an NIR irradiation device equipped with an 800 nm filter (PIS-UHX-NIR; NIPPON P·I Co., Ltd., Tokyo, Japan), each well was irradiated with NIR at an output of 60 mW from the bottom of the six-well plate for 30 min in a constant-temperature room at 37 °C.

#### 4.1.3. Measurement of Medium Temperature

Changes in medium temperature over time were measured using a digital thermometer (UNIQUE MEDICAL, Tokyo, Japan).

#### 4.1.4. Lactate Dehydrogenase (LDH) Release Assay

Cytotoxicity was evaluated using the LDH Cytotoxity Assay Kit (Nacalai Tesque, Kyoto, Japan) as described. Briefly, 100 μL of culture medium was mixed with 100 μL of substrate solution. After incubation under air conditions for 20 min in a light-shielded environment, the reaction was terminated by adding 50 μL of Stop Solution, and the optical densities (ODs) at 490 nm were measured spectrophotometrically. Cells lysed with the Lysis Solution from the kit were used as positive controls.

#### 4.1.5. Real-Time Reverse Transcription PCR (RT-PCR) Analysis

After 6 h, total RNA was extracted from cells using ISOGEN II (Nippon Gene, Osaka, Japan). Reverse transcription was performed using ReverTra Ace^®^ qPCR RT Master Mix (Toyobo, Osaka, Japan) according to the manufacturer’s instructions. Quantitative real-time RT-PCR was performed using the Applied Biosystems 7300 Real-Time PCR System (Applied Biosystems, Carlsbad, CA, USA) with a TaqMan gene expression assay (Applied Biosystems) for *HSP70* (Rn04224718_u1), *ACAN* (Rn00573424_m1), and *ColII* (Rn01637087_m1). Each 20 μL reaction mixture contained 2 μL of cDNA and 10 μL TaqMan^®^ Gene Expression PCR Master Mix (TOYOBO, Osaka, Japan) for the target gene. The amplification protocol consisted of 40 cycles of denaturation at 95 °C for 15 s and annealing and extension at 60 °C for one min. Relative changes in gene expression were determined using the comparative CT method. The 18S ribosomal RNA was used as the internal control [forward primer, 5′-ATGAGTCCACTTTAAATCCTTTAACGA-3′; reverse primer, 5′-CTTTAATATACGCTATTGGAGCTGGAA-3′; probes, 5′-(FAM)ATCCATTGGAGGGCAAGTCTGGTGC(BHQ)-3′]. Each experiment was repeated three times. The results of all experiments showed a similar trend.

#### 4.1.6. HSP70 Inhibition Experiment

An inhibition experiment was conducted with Que to determine whether the enhancement of *ACAN* gene expression was mediated by HSP70. Que (Sigma-Aldrich, St. Louis, MO, USA) was added to DMEM containing 0.05 g/L ICG to a final concentration of 200 μM, was irradiated with NIR, and underwent RT-PCR as described above. To explore other factors influencing chondroprotective effects, the expression of each gene was also measured using the TaqMan gene expression assays for *SOX9* (Rn01751070_m1), *ADAMTS4* (Rn02103282_s1), and *MMP-13* (Rn01448194_m1).

### 4.2. In Vivo Studies

#### 4.2.1. Preparation of Animals

Experiments were conducted using ten-week-old male Wistar rats (Shimizu Laboratory Suppliers, Kyoto, Japan). A total of 42 rats were divided into three groups: a group with articular cartilage isolation (n = 24), a group with ICG injected into the knee joint and NIR irradiation (ICG+NIR group, n = 9), and a group with PBS injected into the knee joint and no NIR irradiation (control group, n = 9). This study was conducted in accordance with the regulations on animal experiments at our facility (Code No. M2020-291).

#### 4.2.2. Intra-Articular Injection of ICG and NIR Irradiation

The irradiation intensity and ICG concentration were preliminarily studied in vitro; the ICG concentration was set to 2.5 g/L, and the irradiation intensity was set to 185 mW. PBS (50 μL) containing 2.5 g/L ICG was injected into the left knee joint of rats that were anesthetized with a mixture of 0.375 mg/kg medetomidine, 2.0 mg/kg midazolam, and 2.5 mg/kg butorphanol and irradiated from the front of the flexed knee joint using an NIR irradiation device (PIS-UHX-NIR) equipped with an 800 nm filter at 185 mW for 1 h. As a control, 50 μL of PBS was injected into the left knee joint, and rats without irradiation were used.

#### 4.2.3. Temperature Measurement in Joint Cavity

A temperature probe (0.5 mm) was inserted into the left knee joint of rats, and the temperature was measured using a digital thermometer (UNIQUE MEDICAL).

#### 4.2.4. Histochemical Analysis

Rats were anesthetized deeply with pentobarbital and euthanized 8 h after NIR irradiation, and the left knee joint was removed for histological evaluation. After fixation with 4% paraformaldehyde (Wako, Osaka, Japan), they were demineralized with 10% EDTA and embedded in paraffin. Sagittal sections 6 μm thick were prepared from the medial center of the knee and stained with hematoxylin–eosin (HE) and Safranin O. For HSP70 immunohistochemistry, paraffin-embedded sections were de-paraffinized in xylene, rehydrated through graded alcohols by immersion, and washed with running water and PBS. Endogenous peroxidase activity was blocked by incubating the sections in 3% H_2_O_2_ in methanol for 15 min. The sections were incubated overnight at 4 °C with mouse monoclonal anti-HSP70 (ab2787; Abcam, Cambridge, UK) at a 1:200 dilution. After extensive washing with PBS, the sections were incubated in Histofine^®^ Simple Stain Rat MAX-PO (NICHIREI BIOSCIENCES INC., Tokyo, Japan) for 30 min at 25 °C. Immunostaining was detected using DAB staining. Counter-staining was performed with Mayer’s hematoxylin. The total number of chondrocytes and the number of HSP70-staining-positive chondrocytes were determined at 40× magnification.

#### 4.2.5. Real-Time RT-PCR Analysis

The articular cartilage was aseptically collected from the left knee and stored in the RNA Protect^®^ Tissue Reagent (Microtech Nichion, Chiba, Japan), frozen in liquid nitrogen, and then crushed using a Cryo-Press CP-100WP (Microtech Nichion, Chiba, Japan). Total RNA was extracted from the crushed sample using ISOGEN II (NIPPON Gene). *HSP70*, *ACAN*, *ColII;*, *SOX9*, *ADAMTS4*, and *MMP-13* gene expressions were normalized using the internal control gene *18rRNA*.

### 4.3. Statistical Analysis

Data are expressed as the mean ± standard deviation. Data were analyzed using EZR (Saitama Medical Center, Jichi Medical University, Saitama, Japan), a graphical user interface in R (The R Foundation for Statistical Computing, Vienna, Austria) [46]. The data were analyzed by *t*-test and one-way analysis of variance, followed by the Tukey–Kramer test for post hoc analysis. *p* < 0.05 was considered to indicate a statistically significant difference.

## 5. Conclusions

This study revealed the usefulness of a thermal stimulation technique using photosensitizers. This method presents a new preventive and therapeutic method based on cartilage metabolism enhancement.

## Data Availability

Data is contained within the article.
